# Maintaining Blood Glucose Levels in Range (70–150 mg/dL) is Difficult in COVID-19 Compared to Non-COVID-19 ICU Patients—A Retrospective Analysis

**DOI:** 10.3390/jcm9113635

**Published:** 2020-11-12

**Authors:** Rajat Kapoor, Lava R. Timsina, Nupur Gupta, Harleen Kaur, Arianna J. Vidger, Abby M. Pollander, Judith Jacobi, Swapnil Khare, Omar Rahman

**Affiliations:** 1 Division of Pulmonary, Critical Care, Sleep and Occupational Medicine, Department of Medicine, Indiana University School of Medicine, Indianapolis, IN 46202, USA; orahman@iuhealth.org; 2 Department of Surgery, Center for Outcomes Research in Surgery, Indiana University School of Medicine, Indianapolis, IN 46202, USA; ltimsina@iu.edu; 3 Division of Nephrology, Department of Medicine, Indiana University School of Medicine, Indianapolis, IN 46202, USA; nugupta@iu.edu; 4 Department of Medicine, Indiana University School of Medicine, Indianapolis, IN 46202, USA; kaur8@iu.edu; 5 Department of Pharmacy, Indiana University Health, Indianapolis, IN 46202, USA; avidger@iuhealth.org (A.J.V.); apollander@iuhealth.org (A.M.P.); 6 Sr. Consultant Visante, Inc., St. Paul, MN 55101, USA; jjmowry426@gmail.com; 7 Division of Endocrinology, Department of Medicine, Indiana University School of Medicine, Indianapolis, IN 46202, USA; khares@iu.edu

**Keywords:** intensive care unit, time in range, COVID-19, non-COVID-19

## Abstract

Beta cell dysfunction is suggested in patients with COVID-19 infections. Poor glycemic control in ICU is associated with poor patient outcomes. This is a single center, retrospective analysis of 562 patients in an intensive care unit from 1 March to 30 April 2020. We review the time in range (70–150 mg/dL) spent by critically ill COVID-19 patients and non-COVID-19 patients, along with the daily insulin use. Ninety-three in the COVID-19 cohort and 469 in the non-COVID-19 cohort were compared for percentage of blood glucose TIR (70–150 mg/dL) and average daily insulin use. The COVID-19 cohort spent significantly less TIR (70–150 mg/dL) compared to the non-COVID-19 cohort (44.4% vs. 68.5%). Daily average insulin use in the COVID-19 cohort was higher (8.37 units versus 6.17 units). ICU COVID-19 patients spent less time in range (70–150 mg/dL) and required higher daily insulin dose. A higher requirement for ventilator and days on ventilator was associated with a lower TIR. Mortality was lower for COVID-19 patients who achieved a higher TIR.

## 1. Introduction

### Background

Uncontrolled hyperglycemia is associated with increased mortality, morbidity, in-hospital stroke mortality, secondary infections, and coronary artery disease in intensive care unit (ICU) patients [1,2,3,4,5,6]. Cortisol release due to stress and cytokine signaling leads to excess hepatic gluconeogenesis, impaired utilization of glucose, and insulin deficiency. Further, withholding outpatient antidiabetic medications, addition of inpatient medication (corticosteroids), and enteral and parenteral nutrition contribute to hyperglycemia [5,7]. Many studies tried to identify optimal blood glucose levels and the tools to achieve them in order to improve mortality within ICU [5,8,9].

The definition of optimal blood glucose control is contentious. The NICE-SUGAR study and meta-analysis linked intensive blood sugar control (<110 mg/dL) with hypoglycemia and higher mortality [9,10]. Lanspa et al. showed that achieving >80% time in range (TIR) of 70−139 mg/dL showed promising outcomes in multi-center ICU patients using a computerized e-Protocol. Achieving this TIR goal was independently associated with lower 30-day mortality in nondiabetic and diabetic with previous good control (HbA1 c ≤ 6.5%), but was not beneficial with prior poor diabetes control [8]. TIR is suggested to be the unifying metric to account for hypoglycemia, glycemic variation, and hyperglycemia events. While it is believed that controlling glucose in critically ill patients is beneficial, the optimal goal for patients with pre-existing poor glucose control is not well known. While the Society of Critical Care Medicine and American Diabetes Association guidelines suggest achieving glucose < 180 mg/dL in critically ill patients on an insulin infusion, lower goals (<150 mg/dL) may be used if a low incidence of hypoglycemia is maintained [5,11]. Insulin administration should be guided by validated protocols, and many have suggested computerized programs for consistency and safety [11].

Severe acute respiratory syndrome coronavirus 2 (SARS-Cov2), causing coronavirus disease 2019 (COVID-19), is a threat to global health. The current understanding of SARS-CoV2 pulmonary pathology is invasion of the respiratory tract and lungs leading to viral pneumonia. The infected patients may develop hypoxic respiratory failure requiring mechanical ventilation, septic shock, along with multi-organ failure and death [12]. Predisposing conditions like type 2 diabetes mellitus along with poor glycemic control, chronic kidney disease, and obesity are associated with severe manifestation of COVID-19 disease [13,14,15]. Hyperglycemia resulting from the inflammatory response, insulin resistance, and pancreatic injury is described in severe COVID-19 infections [16]. Emerging evidence hypothesizes that hyperglycemia may trigger an altered immunologic response in COVID-19 resulting in increased morbidity [17]. The insulin resistance induced by COVID-19 and gluconeogenesis due to critical illness may make glycemic control challenging and potentially impact clinical outcomes. We compared percent time in range of glucose and insulin use as a surrogate for glycemic control amongst COVID-19 and non-COVID-19 ICU patients.

## 2. Materials and Methods

### 2.1. Study Design

The study was a single center retrospective data analysis for patients admitted to 130 ICU beds in the 600-bed Indiana University Health, Methodist Hospital (Indianapolis, IN, USA) from 1 March to 30 April 2020. The study was approved as exempt by the Kuali Coeus IRB (Protocol no. 2004500099). The institutional information technology (IT) team assisted with data extraction and time stamps for analysis.

### 2.2. Patient Selection

All subjects admitted to the ICU were identified based on the location and level of care orders. Patients were admitted to the ICU following an assessment by the primary team on the patient’s clinical condition and risk for imminent worsening. Hospitalist/Intensivists clinical judgement was relied upon for the transfer into or out of the ICU. Patients were excluded if they had an underlying diagnosis of hyperosmolar nonketotic hyperglycemia, diabetic ketoacidosis, and beta-blocker or calcium channel blocker overdose requiring an alternative protocol for insulin therapy.

The patient population was then divided into 2 cohorts—COVID-19-related ICU admission and non-COVID-19-related ICU admission based on the positive COVID-19 RNA PCR from nasopharyngeal–oropharyngeal swab.

### 2.3. Variables

Data were abstracted retrospectively from prospectively collected data in the electronic medical record (Cerner, Kansas City, MO, USA) including demographics, age, admission body mass index, and pre-existing conditions. Pre-existing comorbidities were captured from the provider documentation using the ICD-10-CM coding algorithms [18]. The glycosylated hemoglobin A1C (HbA1C) at the time of admission was used if available, or the most recent values within the previous 3 months as a marker for previous glycemic control. Home diabetic therapy was obtained from the medication reconciliation performed at the time of admission. The pharmacist performs reconciliation via an extensive discussion with the patient (if able), next of kin, healthcare power of attorney, insurance claims, and/or pharmacy fill records. Home diabetic therapy was categorized as—insulin, non-insulin glucose lowering agents (Hypoglycemic agents), and diet-controlled.

Patient’s respiratory status and level of support was reviewed during the ICU stay. Supportive interventions, including invasive ventilation, noninvasive ventilation, high-flow nasal cannula (Vapotherm, Optiflow), and nasal cannula, were documented. Use of proning post intubation, neuromuscular blockade, and extra corporeal membranous oxygenation (ECMO) was documented as separate events. Most patients required several of these interventions at some point and these were counted as unique events. ICU medication administration record (MAR) was reviewed, and use of corticosteroids, vasopressors, and COVID-19-related medications (remdesivir, tociluzumab, hydroxychloroquine) was identified for all patients during ICU stay. Drug administration was only documented in patients with confirmed drug delivery. Medications are scanned at the time of administration in over 90% of doses, and this enhances the accuracy of the medication administration record as a source of data. High-dose ascorbic acid therapy was not used as it is not a standard of care at our facility.

### 2.4. Glucose Management

The decision to order insulin via any route was made by the provider on admission and reevaluated daily. For persistent glucose values greater than 150 mg/dL, patients were started on an insulin infusion or subcutaneous insulin using a correction scale (blood glucose every 4−6 h) plus basal insulin when needed to achieve desired goals. Transition from subcutaneous to intravenous is based on the level of control or variability within the blood sugar levels. We were unable to capture the frequency of transitions between the subcutaneous and intravenous routes.

A computer-based insulin protocol is used for achieving blood glucose level < 150 mg/dL. The centralized insulin dosing software is based on the measurement of blood glucose level, specified insulin sensitivity, carbohydrate intake, and responsiveness to the previous insulin dosing [19,20]. This program is known as the “GlucoStabilizer”. It provides appropriate insulin coverage while minimizing the use of only sliding scale insulin, missed insulin dose adjustments, and calculation errors. The program calculates insulin dosing based on glucose measurements and carbohydrate intake for patients with hyperglycemia of any etiology. This program reminds the timing of glucose level checks and recommends insulin dosing based on the insulin sensitivity factor and carbohydrate ratio ordered by the primary provider team. The subcutaneous GlucoStabilizer program does not optimize its settings based on patients’ blood sugar responses to the insulin dose given. The intravenous GlucoStabilizer program learns and adjusts to meet the changing need of the patient. (The rate of the insulin infusion is calculated by rate = glucose − 60 × multiplier, where the default multiplier = 0.02. The default target blood glucose is 100−150 mg/dL and if after 1 h, the blood glucose is greater than 150 mg/dL, the multiplier increases to 0.03). This program is also equipped to manage the hypoglycemic treatment for the patients. It calculates in “ml” the volume of 50% dextrose solution to be given for blood sugar less than 70 mg/dL. This program has been the lifeline of blood sugar management for the Indiana University health campus for optimizing glycemic management.

The GlucoStabilizer standardizes intravenous and subcutaneous insulin therapy at our institute. Details of this program have been published previously, and it has been associated with high target achievement and low incidence of severe hypoglycemia [21]. We selected a threshold of 85% for the TIR, since our range was slightly higher (70−150 vs. 70−139 mg/dL) compared to the study by Lanspa et al. [8]. We used the range of 100–150 mg/dL, since the standard protocols used within our health care system are built to maintain the blood sugars <150 mg/dL.

Infusion pumps (BD Alaris, Franklin Lakes, NJ, USA) were located in the patient’s room and the GlucoStabilizer program is activated on the bedside computer/monitors on most occasions. The program reminds the bedside nurse to perform blood glucose checks at the recommended frequency (every 4 h, every 6 h (for subcutaneous), or hourly (for intra venous)). Blood glucose was measured using the Accu-Chek Inform meter system (Roche, Indianapolis, IN, USA) on capillary samples, whole blood samples, or with the Abbott i-STAT (Abbott Park, IL, USA) on whole blood, as determined by the bedside nurse. Data were not collected to describe the actual source/methodology. Glucose values are automatically uploaded to the electronic medical records.

Mean daily insulin use for all types and routes of administration was calculated with the use of the MAR time stamp for the insulin administration.

### 2.5. End Points/Outcomes

The primary endpoints were the percentages of time in range (<70, 70−150, 150−250, and >250 mg/dL) and average daily insulin use for patients in the ICU.

The secondary outcome measured was 28-day mortality among the cohorts. We also measured the glucose level variability and peak glucose levels. Mortality, days on ventilator, and respiratory support were compared in both the cohorts among patients with > 85% time in range (70−150 mg/dL) and <85% time in range (70−150 mg/dL).

### 2.6. Statistical Analysis

Descriptive statistics (mean with standard deviations and proportions) were computed to describe the study population using demographic, laboratory, and clinical characteristics. These patient characteristics were compared between COVID-19-positive and non-COVID-19 patients using bivariate Chi-square tests or Fisher’s exact tests for categorical variables and Wilcoxon rank sum tests for non-normally distributed continuous variables. We also grouped the patients using ≥85% vs. <85% of the percentage of times that the glucose level was in the range 70−150 mg/dL to identify the difference in their outcomes among COVID-19 and non-COVID-19 patients using Chi-square, Fisher’s exact, and Wilcoxon-rank tests, as appropriate. Two separate multivariable generalized estimating equations with mortality and glucose in range as the outcome variables and with logit link function, accounting for the correlation of repeated measurements over time with robust standard errors, were created to examine the effect of having COVID-19 infection compared to other critically ill patients admitted to ICU. The multivariable model included demographic, clinical/medical, and laboratory variables. Trends of glucose levels over time for COVID-19 vs. non-COVID-19 patients were computed to track the average time-in-range after days from ICU admission. This line plot was used to examine the number of times that the patients fell within the predefined glucose levels over the period of their ICU stay. Shapiro–Wilk tests were also used to examine the normality of various laboratory variables by the levels of COVID-19 status and we found that these were not normally distributed. Using the kernel density plot for linearity process, we also observed the linearity assumption was not true. Hence, we used a fractional polynomial model to fit the curvilinear (non-Gaussian) pattern of the laboratory variables repeated over time [22,23]. Time-to-event analysis was also performed using log-rank tests to examine the difference in the 28 days survival probability between COVID-19 and non-COVID-19 patients and was portrayed using a Kaplan–Meier curve. All hypothesis tests were done at the 0.05 level of significance using Stata/SE 14.2 [24].

## 3. Results

### 3.1. Baseline Characteristics

A total of 571 unique patients were admitted to ICU in the two months of study duration. Nine patients were excluded based on the exclusion criteria. Five-hundred-sixty-three patients were included in the analysis. Patients were divided into two cohorts based on COVID-19 status. Ninety-three patients were included in the COVID-19 cohort and 469 in the non-COVID-19-related illness cohort. The non-COVID-19 cohort comprised the majority of the patients from medical, surgical, and trauma ICU. Elective cardiovascular surgery and elective neurosurgical procedures were cancelled to maintain optimal resource utilization during the peak COVID surge at the facility. Only patients who required emergency interventions were admitted.

Table 1 described the baseline characteristics of the patient population. The COVID-19 cohort had more African American and Hispanic patients as compared to non-COVID-19 (52.69% versus 28.78%; 12.90% versus 3.84%, *p* <0.001, respectively). The population of Marion County, where our hospital is located, is approximately 29% African American (9.9% in the state of Indiana) [25]. As of 30 April (last day for patient inclusion in the analysis), the state had documented 18,545 cases of confirmed COVID-19, with a cumulative mortality of 1154 [26]. Hospitalizations for COVID-19 have been predominantly in the 50+ year old cohort, consistent with the age group in both cohorts in our study [27,28].

Bivariate descriptive analyses showed no significant differences in age, gender, comorbidities, and prescribed medical therapy for diabetes. However, body mass index of the patients with COVID-19 was higher than the non-COVID-19 cohort (31.15 versus 29.55 kg/m^2^, *p* = 0.0253). Higher frequencies of preexisting chronic kidney disease (32.26% versus 22.39%, *p* = 0.042) occurred in COVID-19 patients as compared to non-COVID-19 patients. Median glycosylated hemoglobin A1C (HbA1C) level on admission was higher in the COVID-19 cohort, suggesting inadequate pre-admission diabetes control (6.8% versus 6.1%, *p* < 0.001). A total of 403 patients (65 COVID-19 and 338 non-COVID-19) had an available HbA1C at the time of admission. A majority of the patients in both cohorts had HbA1C < 7%. A majority of the patients in both cohorts had HbA1C < 7%, as expected with the prevalence of diabetes by history. The Charlson Comorbidity Index compared for the COVID-19 and non-COVID-19 cohorts was similar (*p* = 0.666). Patients with COVID-19 required more aggressive respiratory support in the form of high-flow nasal cannula (HFNC) (53.76% versus 12.79%, *p* < 0.001), and mechanical ventilation (70.97% versus 42.44%, *p* < 0.001) compared to non-COVID-19 patients. Advanced supportive care such as proning, neuromuscular blockade, and extracorporeal membranous oxygenation (ECMO) was more prevalent in the COVID-19 population. Patients with COVID-19 stayed ventilated for a longer duration (9.56 days versus 3.87 days, *p* < 0.001). Supportive and therapeutic medications, such as corticosteroids (61.2% versus 31.5%, *p* < 0.001) and vasopressor (54.8% versus 30.9%, *p* < 0.001), were used more often in patients with COVID-19. Remdesivir and tocilizumab were exclusively used in COVID-19 patients, and hydroxychloroquine was used predominantly in COVID-19 patients (72.0% vs. 1.9%).

### 3.2. Time in Range of Blood Glucose Level and Insulin Utilization

**Figure 1:**
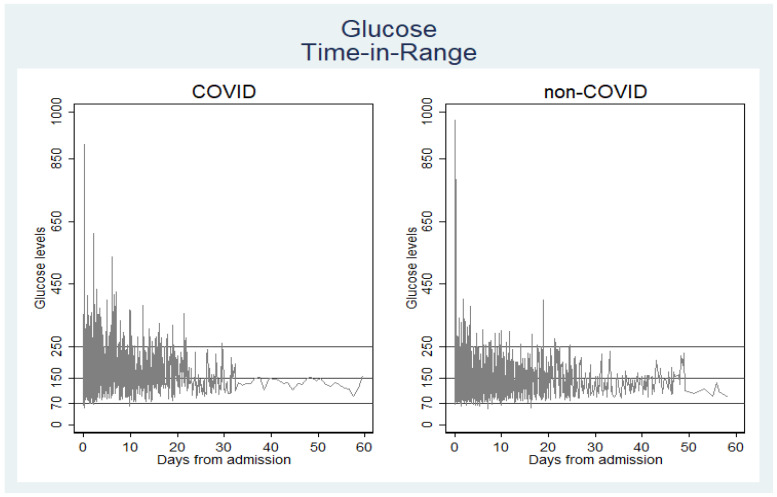
Stagger diagram comparing glucose levels spread over time and range among the COVID-19 and non-COVID-19 study population.

Median number (Interquartile range) of daily blood glucose level checks among COVID-19 was 5 (0–14) and non-COVID-19 was 2 (0–23). Table 2 shows COVID-19 patients spent 44.42% TIR of 70–150mg/dL, 43.48 percent TIR of 151–250 mg/dL, and 11.66 percent TIR of >250 mg/dL (*p* < 0.001). The non-COVID-19 cohort spent 68.52 percent TIR of 70–150 mg/dL. Figure 1 depicts the stagger variations of the glucose levels within the COVID-19 and non-COVID-19 ICU patients. The mean and median blood glucose level in COVID-19 patients was significantly higher compared to non-COVID-19 patients (170.59 and 157 mg/dL vs. 140.37 and 130 mg/dL). Mean and median peak glucose levels were significantly higher in COVID-19 patients in comparison to non-COVID-19 patients (243.07 and 215 mg/dL vs. 179.18 and 160 mg/dL). The glucose check frequency was consistent among both cohorts. Median number (interquartile range) of daily blood glucose level checks among COVID-19 patient was 5 (0–14) and among non-COVID-19 was 2 (0–23). Patients with COVID-19 required higher average daily doses of insulin compared to non-COVID-19 patients (8.37 units versus 6.17 units, *p* < 0.001).

**Figure 2:**
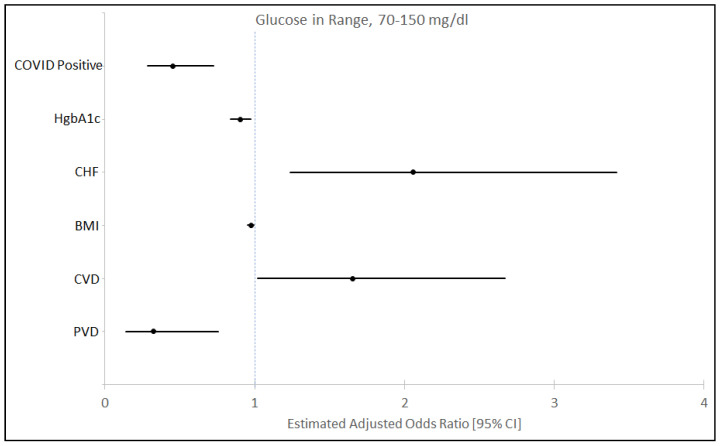
Multivariate analysis with odds ratio (OR) and confidence interval (CI) for time in range (TIR) 70–150 mg/dL in the study population. Other variables adjusted but did not show significance were gender, age, race, myocardial infarction, arrhythmias, chronic lung diseases, and respiratory interventions during the hospital stay and medications like insulin, pressors and steroids. PVD—Peripheral Vascular Disease, CVD—Cerebro Vascular Disease, BMI—Body Mass Index, CHF—Congestive Heart Failure.

A multivariate analysis examined variables associated with ≥ 85% TIR (Figure 2). The COVID-19 status (OR, 0.455; 95% CI, 0.284–0.727), HbA1C (OR, 0.904; 95% CI, 0.839–0.974), BMI (OR, 0.974; 95% CI, 0.954–0.994), and history of peripheral vascular disease (OR, 0.327; 95% CI, 0.141–0.759) were associated with lower odds of having > 85% time in range (70–150 mg/dL). Higher odds of having TIR ≥ 85% were higher with history of congestive heart failure (OR, 1.652; 95% CI, 1.022–2.67) and cerebrovascular disease (OR, 1.652; 95% CI, 1.022–2.67).

Table 3 shows COVID-19 and non-COVID-19 cohorts’ patients with ≥ 85% TIR (70–150 mg/dL) were associated with less days on ventilators (*p* = < 0.001, *p* = < 0.001), respectively. COVID-19 patients requiring more aggressive respiratory support with the use of high-flow nasal cannula (*p* = *0*.009) and mechanical ventilation (*p* = < 0.001) spent < 85% time in range (70–150 mg/dL) during their ICU stay. Patients in both cohorts who required use of neuromuscular blocking agents (paralytics) spent < 85% time in range (COVID-19 *p* = < 0.001 and non-COVID-19 *p* = 0.044). Patients requiring ECMO (COVID-19 *p* = *0*.183, non-COVID-19 *p* = *0*.238) and use of proning (COVID-19 *p* = *0*.704, non-COVID-19 *p* > *0*.99) did not have a significant difference among the patients with >/=85% time in range. Mortality was also noted to be significantly higher in the population with <85% time in range in the non-COVID-19 cohort.

Patients with available HbA1C levels were compared for time in range (70–150 mg/dL). The majority of the patients in all three subgroups (HbA1C < 7, 7.1–8.0, and > 8.1%) spent < 85% of time in range. (Table 4).

### 3.3. Mortality

**Figure 3:**
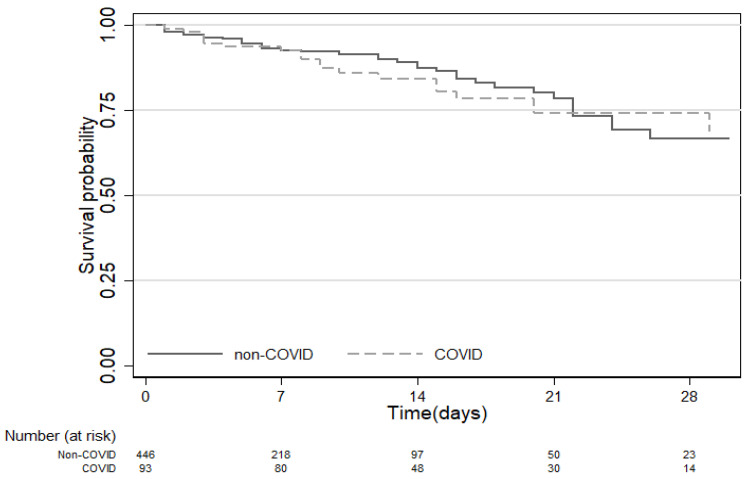
Kaplan–Meier curve comparing 28-day mortality of COVID-19 and non-COVID-19 ICU patients.

**Figure 4:**
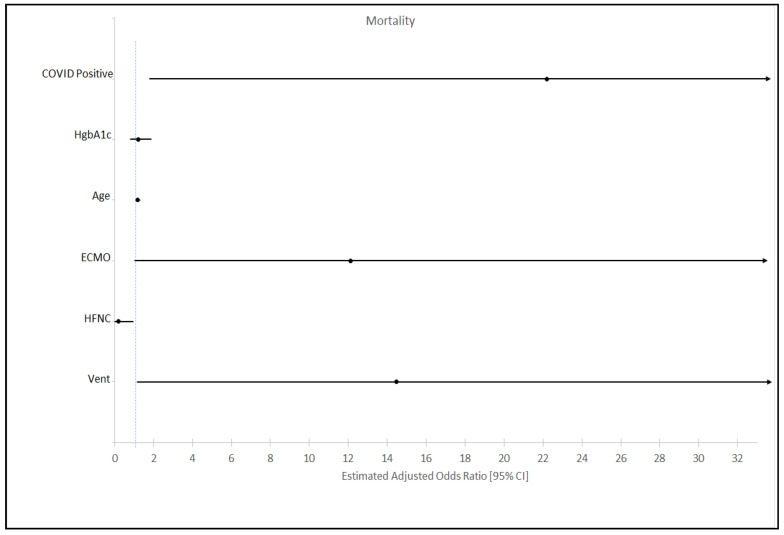
Multivariate analysis with odds ratio (OR) and confidence interval (CI) for mortality in the study population. Other variables adjusted but did not show statistical significance were glucose levels, gender, race, underlying comorbid conditions, proning, paralytics, insulin use, corticosteroids, and vasopressors.

Among the COVID-19 patients, there was no mortality difference among patients ≥ 85% of the TIR (*p* = *0*.085) (Table 3). Mortality difference was identified in the non-COVID-19 cohort among patients ≥ 85% of the TIR versus < 85% of the TIR (*p* = *0*.046). The 28-day non-adjusted mortality among COVID-19 patients was higher than observed in non-COVID-19 patients and trended towards significance (21.51% vs. 13.86%, *p* = 0.06). The Kaplan–Meier plot demonstrated that the 28 days survival probability was not significantly different (Figure 3). Multivariate analysis showed higher odds for mortality (Figure 4) with underlying COVID-19 diagnosis (OR, 22.199; 95% CI, 1.795–274.601), age (OR, 1.187; 95% CI, 1.061–1.328), use of ECMO (OR, 12.132; 95% CI, 1.029–143.02), and mechanical ventilation (OR, 14.458; 95% CI, 1.164–179.644). Use of HFNC was associated with reduced odds ratio (OR, 0.183; 95% CI, 0.035–0.955) for mortality.

## 4. Discussion

Our study identifies COVID-19 ICU patients spent significantly less TIR (70–150 mg/dL) and utilized higher average daily insulin as compared to non-COVID-19 ICU patients. Charlson Comorbidity index was used as a surrogate for defining risk of patient mortality and was similar for both cohorts. While the comorbidity index did not show a difference, these findings are suggestive of more difficult to control blood glucose levels in critically ill COVID-19 infection. Patients with COVID-19 also had significantly higher blood glucose levels (both mean and median) compared to non-COVID-19 patients. Less time spent in range for BG (70–150 mg/dL) was associated with increased utilization of a ventilator and prolonged duration of mechanical ventilation, suggesting severe disease. Higher severity of illness could potentially contribute to variations in glucose levels. We did not compare the severity of these patients using the APACHE or SOFA score, since all the data points were not available for calculation. Multivariate analysis suggested that the presence of COVID-19 infection played a significant role in inability to maintain blood glucose levels in range 70–150 mg/dL.

COVID-19 patients with type II diabetes mellitus are more severe and critically ill on initial presentation [14,15,29,30]. Zhu et al. reported improved outcomes in COVID-19 patients with well-controlled type II diabetes mellitus [31]. The risk of mortality is higher in the uncontrolled diabetes mellitus II subgroup based on a British cohort of 5693 patients. HbA1C of 7.5% or higher has been associated with increased in-hospital mortality within COVID-19 patients [32]. On the contrary, our study shows no 28-day mortality difference between the two cohorts despite higher baseline HbA1C, a surrogate for uncontrolled type II DM, most likely since the average HbA1C did not reach the threshold of 7.5%. Mortality among patients with >85% TIR (70–150 mg/dL) in non-COVID-19 patients was better compared to non-COVID-19 patients with <85% TIR, which is consistent with published evidence, even though we had higher percentage (85% instead of 80%) and higher range (150 mg/dL instead of 139 mg/dL) [8]. The study by Zhu et al. reported inadequately controlled diabetes mellitus was associated with increased mortality [31]. A possible explanation for this observation is the study population. They included the entire hospitalized population, while ours was only limited to the ICU patients. The population reviewed in our study had a similar Charlson Comorbidity Index and a similar frequency of daily blood glucose checks. The former is suggestive of similar patient risk factors while the latter is suggestive of similar patient care and protocol follow up. The concern of reduced frequency of glucose checks being a possible risk factor for poor glycemic control is mitigated by the similar median and interquartile range.

A direct effect of SARS-CoV-2 on pancreatic β-cell function and survival has been suggested, causing worsening rapid and severe deterioration of metabolic control in people with pre-existing diabetes or leading to the development of new-onset diabetes [14]. Angiotensin-converting enzyme 2 (ACE 2) is potentially a crucial molecular link between COVID-19 severity and insulin resistance. ACE 2 is extensively present on the pancreatic beta cells [33] and the ligand through which coronaviruses such as SARS CoV-2 binds to its target cells [34]. Inhibition/blockage of ACE 2 causes a significant increase in angiotensin 2 and hyper-reactivity of the renin–angiotensin–aldosterone system, causing increased oxidative stress and reduced insulin sensitivity [35]. Our findings support this hypothesis as a significantly lower percentage of COVID-19 patients spent TIR of BG 70–150 mg/dL and higher time in > 250 mg/dL. Further, the average daily insulin dose was significantly higher in the COVID-19 cohort. This gives credence to the hypothesis of inherent insulin resistance within the patients affected by COVID-19, regardless of illness severity [16]. The alterations in the post receptor insulin signaling cascades result in the development of insulin resistance [36].

Higher glycemic variability along with more frequent hypoglycemia contributes to increased mortality in previous studies [8]. Our study did not report any association of mortality with TIR, possibly due to smaller sample size and less frequent hypoglycemia in both cohorts. This recapitulates the risk of increased mortality with severe hypoglycemia. Additionally, greater > 85% TIR was associated with lesser utilization and duration of ventilation. Hyperglycemia results in increased glucose concentration in epithelial secretion, disrupting the defense capacity of the airway epithelia, thus prolonging the duration of ventilation [37]. Another important confounder potentially is the presence of undiagnosed/unrecognized diabetes. HbA1C levels were not recorded in all patients, thus it is plausible that the COVID19 group had more patients with unrecognized diabetes and this contributed to the observed differences in glycemic control.

A recent study from Italy comparing hyperglycemia control in critically ill COVID-19 patients with pre-existing DM associated hyperglycemia without insulin infusion with higher risk of severe disease [38]. Although, the severity of disease was described by chest CT images. This study signals towards correlation of intensive glucose monitoring and aggressive insulin regimen to maintain TIR (70–150 mg/dl) with improved ICU outcomes in COVID-19 populations. Although, the causal association of hyperglycemia and severity of disease remains unanswered. The unwanted consequence of intensive regimen is hypoglycemia. IV insulin infusion necessitates frequent glucose monitoring, a challenging task due to isolation and personal protective equipment requirements. Continuous Glucose Monitoring (CGM) devices present a viable solution for frequent monitoring in this clinical scenario [39].

### Limitations

Our study has several limitations. This is a retrospective, cohort-based, single center study. Given the design of the study, where outcomes were already observed in this chart review study, post hoc power analysis will not add much. While a priori power calculation would be an indispensable component of a clinical study, post hoc power analysis of a study, when all eligible subjects are pooled in a study and where outcomes are already observed, will be conceptually flawed and analytically misleading [40,41]. To overcome this limitation, the figures with the results from the multivariable analysis presents the confidence interval of the estimates.

We did not actively monitor the patient’s response to insulin dosing and calculate the insulin resistance pattern using the HOMA or the QUICKI methods [42,43]. We did not collect the SOFA or APACHE score for the patient population. The study was not powered to capture mortality benefit from higher time spent in range (70–150 mg/dL). Even though we noted a trend towards improved mortality, it did not reach predefined statistical significance (*p* < 0.05). The proportion of medical, surgical, and trauma ICU patients within the non-COVID-19 cohort is not available. Trauma and elective surgeries were at a minimum during the imposed lockdown due to the COVID-19 surge. Multiple physicians directed insulin dosing and insulin intravenous infusion/subcutaneous transitions were not standardized. This was difficult to capture in the data analysis. We acknowledge the possible limitation that some patients may have been newly diagnosed with diabetes mellitus during their admission and hence, were not identified as diabetic in the pre-existing diagnosis, although this knowledge would not have altered our treatment strategies.

## 5. Conclusions

The study identifies the difficulty of blood glucose level control in critically ill COVID-19 patients. A higher proportion of COVID-19 patients spent <85% time in range, utilized more insulin per day compared to the non-COVID-19 ICU patients. The findings confirm the difficulty in maintaining blood glucose levels in range and hypothesizes the presence of insulin resistance within critically ill COVID-19 patients. Intensified insulin dosing along with more frequent BG monitoring or potentially using continuous glucose monitoring devices varied from non-COVID-19 patients could assist in maintaining adequate time in the range of blood glucose level (70–150 mg/dL) and thus, improve ICU outcomes.

## Figures and Tables

**Table 1:** Baseline characteristics, respiratory support, and medication interventions.

Baseline Characteristics	In Sample (562)	COVID-19 (93)	Non-COVID-19 (469)	*p*-Value
Age (Years) median (IQR) *^a^*	59.5 (47–69)	61 (51–69)	59 (47–69)	0.2844
Sex—Male (%)	316 (56.23)	50 (53.76)	266 (56.72)	0.6
Body Mass Index kg/m^2^—Median (IQR) ^*a*^	29.70 (24.95–36)	31.15 (26.8–36.9)	29.55 (24.65–35.2)	0.0253
**Race *n*(%)**				
Caucasian	334 (59.43)	28 (30.11)	306 (65.25)	
African American	184 (32.74)	49 (52.69)	135 (28.78)	
Other	14 (2.49)	4 (4.30)	10 (2.13)	<0.001
Hispanic	30 (5.34)	12 (12.90)	18 (3.84)	
**Comorbidities *n*(%)**				
Diabetes Mellitus	192	37 (39.78)	155 (33.05)	0.211
Hyperlipidemia	65 (11.57)	11 (11.83)	54 (11.51)	0.931
Stroke/Cerebrovascular disease *^b^*	57 (10.14)	3 (3.23)	54 (11.51)	0.014
Chronic Kidney Disease	135 (24.02)	30 (32.26)	105 (22.39)	0.042
Coronary Artery Disease *^b^*	15 (2.67)	0 (0)	15 (3.20)	0.149
Congestive Heart Failure	77 (13.70)	8 (8.60)	69 (14.71)	0.117
Arrhythmia	104 (18.51)	12 (12.90)	92 (19.62)	0.128
Chronic Lung disease	139 (24.73)	25 (26.88)	114 (24.31)	0.599
Charlson Comorbidity Index Score				0.666
0	166 (29.53)	142 (30.28)	24 (25.81)	
1–3	317 (56.40)	261 (55.65)	56 (60.22)	
4+	79 (14.03)	66 (14.07)	13 (13.98)	
**DM Tx (home meds)**				
Diet Control (%)	43 (7.65)	12 (12.90)	31 (6.61)	0.037
Non-insulin Hypoglycemic Agents (%)	65 (11.57)	13 (13.98)	52 (11.09)	0.426
Insulin (%)	138 (24.56)	22 (23.66)	116 (24.73)	0.825
HbA1C (*n* = 403) median (IQR) *^a^*	6.2 (5.7–7.2)	6.8 (6–8)	6.1 (5.6–7.1)	<0.001
<7% *n* (%)	288 (51.24)	36 (38.70)	252 (53.73)	
7.1–8% *n* (%)	47 (8.36)	13 (13.97)	34 (7.24)	
>8.1% *n* (%)	68 (12.09)	16 (17.20)	52 (11.08)	
**Respiratory Intervention *n*(%)**				
Nasal Cannula	385 (68.51)	78 (83.87)	307 (65.46)	<0.001
High-Flow Nasal Cannula	110 (19.57)	50 (53.76)	60 (12.79)	<0.001
Non-Invasive Ventilation	66 (11.74)	6 (6.45)	60 (12.79)	0.083
Ventilator	264 (46.98)	66 (70.97)	198 (42.44)	<0.001
Proning	35 (6.23)	25 (26.88)	10 (2.13)	<0.001
Paralytics	112 (19.93)	52 (55.91)	60 (12.79)	<0.001
ECMO	13 (2.31)	7 (7.53)	6 (1.28)	<0.001
Days of Ventilator Mean (SD) *^a^*	4.81 (11.76)	9.56 (9.98)	3.87 (11.86)	<0.001
**Medications *n*(%)**				
Steroids	205 (36.48)	57 (61.29)	148 (31.56)	<0.001
Pressors	196 (34.88)	51 (54.84)	145 (30.92)	<0.001
Remdesivir	4 (0.71)	4 (4.30)	0 (0)	<0.001
Tocilizumab ^*b*^	4 (0.71)	4 (4.30)	0 (0)	0.001
Hydroxychloroquine *^b^*	76 (13.52)	67 (72.04)	9 (1.92)	<0.001

*^a^* Wilcoxon Rank-Sum (Mann–Whitney) test, *^b^* Fisher’s exact test.

**Table 2:** Outcomes data.

Outcome	In Sample (562)	COVID-19 (93)	Non-COVID-19 (469)	*p*-Value
**Insulin use (daily average) *^a^***	7.63 (4.65)	8.37 (4.08)	6.17 (5.30)	<0.001
**Glucose Time in Range (%)**				
<70 mg/dL	0.44	0.44	0.44	
70–150 mg/dL	60.13	44.42	68.52	
151–250 mg/dL	33.31	43.48	27.88	<0.001
>250 mg/dL	6.12	11.66	3.16	
**Glucose mg/dL**				
Mean (SD)	150.89 (60.51)	170.59 (66.60)	140.37 (54.13)	<0.001
Median (IQR) *^a^*	136 (112–174)	157 (124–205)	130 (107–159)	
Coefficient of Variation in Glucose level	0.40	0.39	0.38	
**Peak Glucose mg/dL**				
Mean (SD)	190.31 (98.79)	243.07 (122.62)	179.18 (89.25)	<0.001
Median (IQR) *^a^*	164 (130–218.5)	215 (146–323)	160 (128–201.5)	
**Mortality *n* (%)**	85 (15.12)	20 (21.51)	65 (13.86)	0.06

*^a^* Wilcoxon Rank-Sum (Mann–Whitney) test.

**Table 3:** Respiratory support and outcome compared with glycemic control (*n* = 93).

Outcome	In Sample COVID-19 *n* = 93(%) Non-COVID-19 *n* = 469(%)	>/=85% in Range: 70–150 mg/dL COVID-19 Non-COVID-19	<85% in Range: 70–150 mg/dL COVID-19 NON-COVID-19	*p*-Value
**Mortality *n* (%) ^a^**				
COVID-19	20 (21.51)	2 (10)	18 (90)	0.085
Non-COVID-19	65 (13.86)	21 (32.3)	44 (67.69)	0.046
**Days of Ventilator Mean (SD) ^b^**				
COVID-19	9.56 (9.98)	1.84 (3.59)	12.40 (10.09)	<0.001
Non-COVID-19	3.87 (11.86)	2.12 (5.48)	5.22 (14.93)	<0.001
**High Flow Nasal Cannula *n* (%)**				
COVID-19	50 (53.76)	19 (38)	31 (62)	0.009
Non-COVID-19	60 (12.79)	24 (40)	36 (60)	0.535
**Ventilator *n* (%)**				
COVID-19	66 (70.97)	8 (12.12)	58 (87.87)	<0.001
Non-COVID-19	198 (42.22)	72 (36.36)	126 (63.63)	0.006
**Proning *n* (%) ^a^**				
COVID-19	25 (26.88)	6 (24)	19 (76)	0.704
Non-COVID-19	10 (2.13)	4 (40)	6 (60)	>0.99
**Paralytics *n* (%)**				
COVID-19	52 (55.91)	6 (11.53)	46 (88.46)	<0.001
Non-COVID-19	60 (12.79)	19 (31.66)	41 (69.34)	0.044
**ECMO *n* (%) ^a^**				
COVID-19	7 (7.53)	0 (0)	7 (100)	0.183
Non-COVID-19	6 (1.28)	1 (16.67)	5 (83.33)	0.238

^a^ Fisher’s exact test, ^b^ Wilcoxon rank sum (Mann–Whitney) test. Percentages in column 2 are calculated from the in-sample total number vertically. Column 3 and 4 percentages are calculated horizontally based on the *n* from Column 2.

**Table 4:** Admission/preadmission HbA1C effect on time in range (70–150 mg/dL).

HbA1C	In Sample COVID-19 *n* = 65(%) non-COVID-19 *n* = 338(%)	>/=85% in Range: 70–150 mg/dL COVID-19 non-COVID-19	<85% in Range: 70–150 mg/dL COVID-19 non-COVID-19	*p*-Value
**<=7%**				
COVID-19 *n* (%)	36 (55.38)	7 (19.44)	29 (80.56)	0.014
Non-COVID-19 *n* (%)	252 (74.56)	103 (40.87)	149 (59.12)	<0.001
**7.1–8.0%**				
COVID-19 n (%)	13 (20.00)	0 (0)	13 (100)	0.329
Non-COVID-19 n (%)	34 (10.06)	3 (8.82)	31 (91.18)	0.002
**>=8.1%**				
COVID-19 *n* (%)	16 (24.62)	0 (0)	16 (100)	0.18
Non-COVID-19 *n* (%)	52 (15.38)	4 (7.69)	48 (92.31)	<0.001

Percentages in column 2 are calculated from the in-sample total number vertically. Column 3 and 4 percentages are calculated horizontally based on the *n* from Column 2.

## Abbreviations

TIR	time in range;
COVID-19	Coronavirus disease 2019;
non-COVID-19	non-coronavirus-related disease 2019;
BG	blood glucose; ICU—intensive care unit;
CI	confidence interval;
OR	odds ratio;
ACE2	angiotensin converting enzyme 2.
